# Effect of Tempering Treatment on Microstructural Evolution and Mechanical Behavior of Heavy-Wall Heat Induction Seamless Bend Pipe

**DOI:** 10.3390/ma15010259

**Published:** 2021-12-30

**Authors:** Juntai Hu, Yu Liu, Ge Wang, Qiang Li, Jianyang Wen, Lijun Yan, Shibo Chen, Yunlong Gu

**Affiliations:** 1 National Engineering Research Center for Equipment and Technology of Cold Strip Rolling, Yanshan University, Qinhuangdao 066004, China; kle1129@163.com (J.H.); wangge@ysu.edu.cn (G.W.); wenjy1202@163.com (J.W.); chen1553931107@163.com (S.C.); 2 China Petroleum Pipeline Engineering Corp, Langfang 065000, China; yjyliuyu@cnpc.com.cn (Y.L.); cppmyanlj@cnpc.com.cn (L.Y.); guyunlong@cnpc.com.cn (Y.G.); 3 School of Materials Science and Engineering, Hebei University of Technology, Tianjin 300130, China

**Keywords:** seamless bend pipe, tempering, microstructure, yield behavior, work hardening

## Abstract

In this paper, the microstructure and mechanical properties of heavy-wall seamless bend pipe after quenching at different tempering temperatures, including 550 °C, 600 °C, 650 °C, and 700 °C, were studied. Microstructure and dislocations observations were characterized by means of an optical microscope, a scanning electron microscope, a transmission electron microscope, and X-ray diffraction. As the tempering temperature increases, the dislocation density in the test steel gradually decreases, and the precipitation behavior of (Nb, V)(C, N) increases. The sample tempered at 650 °C exhibits a granular bainite structure with a dislocation cell structure and a large number of smaller precipitates. The yield platforms of tempered samples at 650 °C and 700 °C are attributed to the pinning effect of the Cottrell atmosphere on dislocations. The sample tempered at 650 °C not only presents the highest strength, but also the highest uniform elongation, which is attributed to the higher strain-hardening rate and instantaneous work-hardening index. This is closely related to the multiplication of dislocations, the interaction between dislocations and dislocations, and the interaction between dislocations and precipitates during plastic deformation of the 650 °C-tempered samples with low dislocation density, which delays the occurrence of necking.

## 1. Introduction

Most oil and natural gas production areas are located in remote mountainous areas, deserts, oceans, and other special areas. Pipeline transportation is the most efficient, economical, and safe transportation method for oil and gas transportation. High-pressure, large-diameter, large-throughput, and long-distance transportation have become the inevitable development trend of natural gas pipelines. In order to improve the efficiency of pipeline transportation, reduce the cost of pipeline construction, save steel, and meet the requirements of increasing transportation pressures of the pipeline engineering, it is necessary to improve the grade of pipeline steel and reduce the wall thickness of steel pipe, so as to put forward higher requirements for pipeline steel. Pipeline steel, as the balance body of strength, toughness, and plasticity, should not only have high strength and toughness, but also have good plasticity [1,2,3,4,5,6]. In addition, excellent weldability should be taken into account. Pipeline steel has become the most dynamic and important branch in the field of high-strength low-alloy steel (HSLA) and micro-alloyed steel [7,8,9,10,11]. Defects in crystal structure, including dislocations, solid solution atoms, precipitates, and grain boundaries, are widely accepted as obstacles to dislocation movement. These factors correspond to the corresponding strengthening mechanisms, including dislocation strengthening, solid solution strengthening, precipitation strengthening, and grain boundary strengthening [8,11]. A variety of control methods based on these strengthening mechanisms has been widely proposed to enhance the strength of high-strength low-alloy steel. However, the realization of high strength is usually at the expense of toughness and plasticity. The thermo-mechanical control process (TMCP) is an effective method that combines controlled rolling and controlled cooling technology to obtain excellent comprehensive mechanical properties by regulating the formation of microstructure during thermal deformation [11,12]. Due to low carbon and microalloying, combined with different TMCP conditions, the microstructure of high-strength pipeline steel usually contains different microstructure components, such as polygonal ferrite (PF), quasi-polygonal ferrite (QF), acicular ferrite (AF), bainite ferrite (BF), and martensite–austenite (MA) constituents, forming a complex mixed microstructure with different characteristics. The addition of micro-alloying elements, such as Mn, Mo, Cr, Ni, V, Nb, and Ti, can help to obtain ideal microstructure and mechanical properties [12,13,14].

Considering the complex geological conditions and the preferential change in the laying direction of the pipeline, the bend pipe, with its important functions, can effectively buffer the tensile and compressive stress and torque direction applied on the pipeline [15,16]. At present, the processing technology, combining induction heating, bending, and cooling, namely, the induction heating bending method, is the most advanced forming method for large-diameter and thick-walled bend pipe, and has become the main production technology of bend pipes for oil and gas transportation. The tempering process is the last process of hot bending production; its main purpose is to improve the microstructure and toughness of bend pipe, and to eliminate the residual stress of hot bend pipe. Usually, the same grade steel pipes used to produce hot bend pipes were obtained by TMCP technology in order to achieve the combination of high strength and toughness. In the hot bending process, the parent pipe was heating at high temperature, cooled rapidly, deformed and tempered subsequently. The complex process makes the microstructure and performance evolution of the steel pipe more complex. When the steel pipe was rapidly heated above Ac_3_ temperature, the microstructure and properties formed by TMCP will change greatly [17,18]. From the point of view of bending production technology, large-diameter bend pipe is usually produced by welded pipe. Although the weld is in the neutral deformation zone, the weld and heat-affected zone are also subjected to the same heat treatment process as the pipe body [19]. The microstructure evolution of the weld and heat-affected zone is completely different from that of the parent pipe, which will directly affect the final performance of the bend pipe, and may deteriorate the mechanical properties of the welded heat-affected zone of the bend pipe. It is necessary to master the influence of the hot bending process and the tempering process on the microstructure and properties of pipeline steel to ensure the reliability of the bend pipe in service. Therefore, it is urgent to develop a new type of large-diameter and thick-walled seamless bend pipes with high strength and toughness, which has become a new direction of bend pipe development. It can effectively balance the contradiction between the induction heating bending process and the deterioration of mechanical properties in welding the heat-affected zone. The seamless bend pipe, which has undergone modulation treatment, consisting of induction heating quenching and tempering, avoids the adverse effects on the microstructure and mechanical properties of parent pipe matrix obtained by the TMCP process.

Work-hardening behavior is an important means to investigate the mechanical behavior of materials, which determines the mechanical properties of materials. Under tensile condition, when the work-hardening index is high, the stress rises rapidly and quickly reaches the tensile limit, and then the necking occurs at a point until the fracture occurs. Under complex engineering conditions, the high hardening index will promote the surrounding materials to coordinate the large deformation displacement of the pipeline when the pipeline is subjected to large plastic deformation locally. There is a correlation between the work-hardening characteristics of the material and the plastic elongation. Therefore, work hardening is the core issue of plastic deformation research of pipeline steel, and improving the work-hardening index of pipe is an effective way to improve the deformation capacity of pipeline steel.

A large number of studies have focused on the effects of induction-bending parameters and tempering temperature on microstructure and mechanical properties. Wang et al. [16] reported a new design of X80 steel for thick-walled thermal induction bend pipe, and investigated the effects of hot bending parameters (including heating temperature, cooling rate, tempering temperature) on microstructure and mechanical properties. Zhang et al. [20] studied the effect of tempering temperature on the microstructure and properties of X80 steel-grade hot bend pipe. The results show that with the increase in tempering temperature, the strength decreases, which is attributed to the broadening of bainite ferrite and granular bainite laths, and the decrease in the precipitation-strengthening effect. The evolution of dislocation substructure, the reduction in the pinning effect of carbonitride on dislocation and sub-grain boundary, and the decomposition of MA constituents are beneficial to improving toughness. Zhou et al. [17] reported the effect of the tempering process at different temperatures on the microstructure and mechanical properties of thick-walled bend pipes produced by the ultra-fast cooling technology, which systematically expounded the relationship between strength toughness and dislocation, misorientation angle, effective grain size, as well as microcrack propagation. However, it is regrettable that few articles focus on the microstructure evolution and mechanical behavior of thick-walled seamless bend pipes after tempering treatment.

This paper mainly studied the effects of different tempering temperatures on the microstructure and mechanical properties of thick-walled heat induction seamless bend pipes. Multi-scale characterization involves OM, SEM, TEM, and XRD to reveal the microstructure. The effects of different tempering temperatures on the yield behavior, work-hardening behavior and uniform plastic deformation of seamless bend pipe, and the relationship between strengthening mechanism and work-hardening behavior were systematically discussed.

## 2. Experiment

### 2.1. Materials and Process

The material investigated was an X80 × D1422 mm heavy-wall heat induction bend pipe with an outer diameter of 1422 mm, a wall thickness of 38.5 mm, and a pipe length of 6220 mm. The chemical composition of the investigated seamless bend pipe is listed in Table 1. The composite strengthening and toughening mechanism of adding Nb + Ti + V can be precipitated in the dislocation or ferrite matrix, which effectively refines the grain and improves the strength.

**Figure 1:**
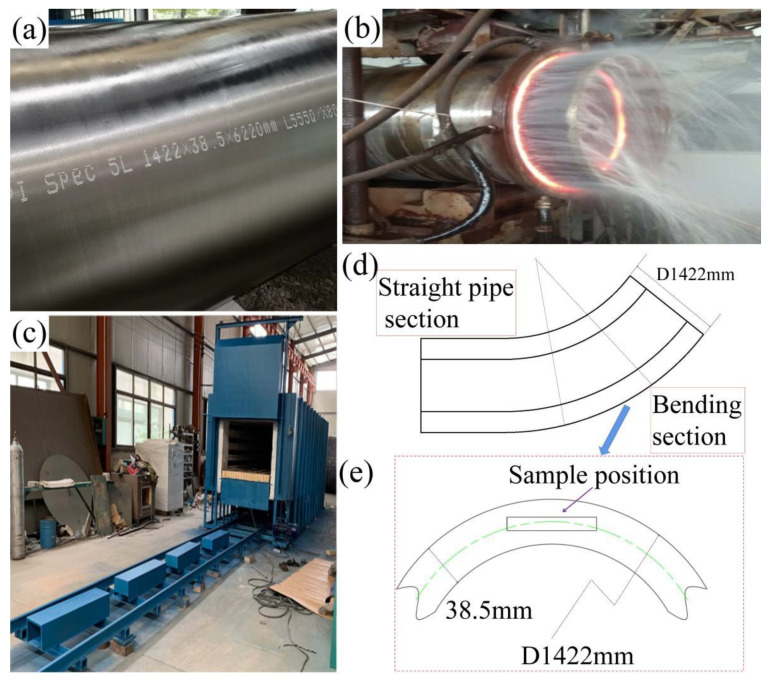
(**a**) parent pipe of seamless bend, (**b**) heat induction bending process, (**c**) RT2-9 bogie-hearth resistance furnace used for tempering, (**d**) the schematic diagram of the heat-induced bending process, and (**e**) the location of the test sample.

The rolling process of the parent pipe includes heating the electroslag remelting blank, oblique rolling piercing, cooling in a cooling bed, and so on. Figure 1a shows the parent pipe of seamless bend. Figure 1b shows the heat induction bending process of X80 heat-induced seamless bend pipe and the process parameters are shown in Table 2. Figure 1c shows the RT2-9 trolley resistance furnace (produced by Henan Tianli Thermal Equipment Co., Ltd., Xinxiang, China) used for tempering. It controls the ambient temperature by thermocouple, which can effectively ensure the homogenization of temperature. The different tempering temperatures are applied to the X80 seamless bend pipe in this study, including 550 °C, 600 °C, 650 °C, and 700 °C. Then, the bend pipe is air-cooled to room temperature outside the furnace. The samples for microstructure observations and tensile tests were taken from the middle position of the thick-walled seamless bend pipe along the wall thickness direction, as shown in Figure 1d,e.

The sample for determining continuous cooling transformation (CCT) diagrams of the X80 heavy-wall heat induction seamless bend pipe steel requires a cylindrical rod with a length of 90 mm and a diameter of 6 mm. With the help of Gleeble 3500 in a vacuum state, the sample is austenitized at 1000 °C for 150 s, and then directly cooled to room temperature at a series of constant cooling rates ranging from 1 °C/s to 60 °C/s. The temperature expansion curve of the sample during the cooling process was recorded, and the metallographic morphology was observed.

### 2.2. Microstructural Investigation

Samples for metallographic observation were prepared by wire-cut electrical discharge machining, mechanical grinding, and polishing, before being etched with a misture solution of 4% HNO_3_ and 96% ethly alcohol. The microstructures before and after heat treatments were investigated by an optical microscope (OM) and a Zeiss Auriga field emission scanning electron microscope (FE-SEM). For further microstructural analysis, transmission electron microscopy (TEM) observation was carried out by a JEM-2100(HR) TEM operated at 200 kV. The samples for TEM observation were prepared by cutting thin wafers from small coupons and grinding them to 50–60 μm. Discs with a diameter of about 3 mm were punched from the wafers and twin-jet electro-polished in a solution containing 90% glacial acetic acid and 10% perchloric acid at 50 V/−30 °C for approximately 2 min. X-ray diffraction (XRD) analysis was carried out to determine the total dislocation density (ρ). The XRD samples were examined over a 2θ range from 40–110° with a step measurement of 0.02° and a scanning speed of 2°/min in a PANalytical X’Pert Pro X-ray diffractometer with Cu-Kα radiation (λ = 0.1506 nm), operated at 150 mA and 40 V using a graphite monochromator.

### 2.3. Mechanical Properties Testing

In order to assess the comprehensive mechanical properties, the tensile test was performed. The specimens for tensile test samples were cut from the middle of the tested bend pipe along wall thickness direction. The longitudinal direction of the tensile specimens is parallel to the transverse direction of the bend pipe. For tensile tests, specimens with round cross-section of 6 mm in diameter and 25 mm of original gauge length were machined, according to the ASTM E8-2016 standard. Tensile tests were conducted at ambient temperature on an 810 Materials Testing System (MTS810) of 250 kN capacity with a strain rate of 1.3 × 10^−3^/s. All tensile samples were measured repeatedly three times, and the average value was taken. An extensometer with a gauge length of 25 mm was used to record the engineering strain during the tensile test. The hardness of the specimen was measured on the HVS-1000 Vickers pyramidal indenter with a test load of 10 kg. Each sample is measured eight times, and the average value is taken as the hardness value.

## 3. Results

### 3.1. Microstrutural Characteristics

#### 3.1.1. Microstructural Evolution

**Figure 2:**
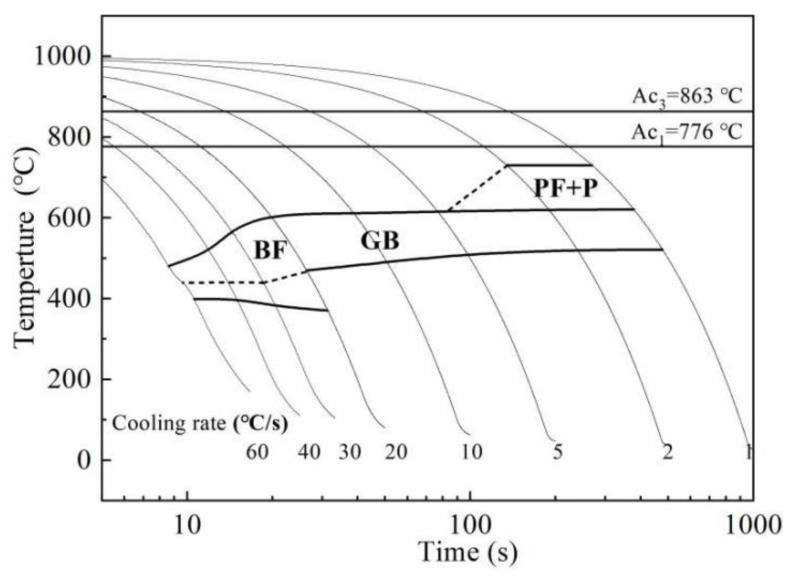
Continuous cooling transformation diagram of seamless pipe steel.

According to the dilatometry experiment, the starting temperature (Ac_1_) and the completion temperature (Ac_3_) for austenitization of the alloy steel were 776 °C and 863 °C, respectively. According to the observation of the phase transition point determined by the expansion method and the metallographic structure, Figure 2 presents the CCT diagram. When the cooling rate is less than 5 °C/s, the austenite is mainly transformed into ferrite and pearlite; when the cooling rate is 5 °C/s to 20 °C/s, the austenite is mainly transformed into granular bainite; and when the cooling rate is greater than 20 °C/s, the austenite is mainly transformed into lath bainite.

**Figure 3:**
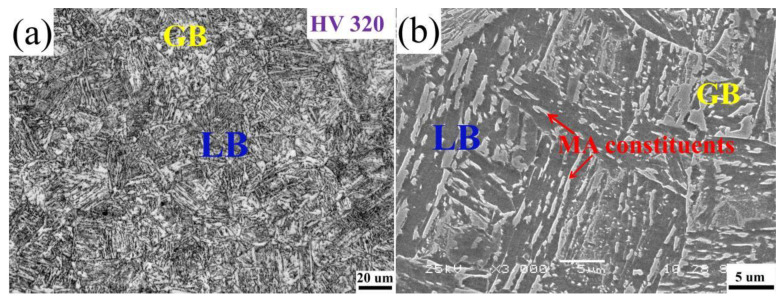
The OM (**a**) and SEM (**b**) of micrographs of as-quenched samples of heavy-wall heat Induction seamless bend pipe.

Figure 3 shows the optical and scanning electron microscope photographs of the investigated steel after quenching, respectively. It shows that the microstructure was made up of mainly lath bainite (LB), very little granular bainite (GB), and some martensite–austenite constituents (MA constituents). The original austenite grain boundary was clearly visible. After heating to austenitizing, during the cooling process, austenite transformed into granular bainite and lath bainite by mixed shear and diffusion. The transformation from austenite to granular bainite occurs before lath bainite, and the nucleation is mainly at the austenite boundary. With the further decrease in temperature, lath bainite is mainly formed within or at austenite grain boundaries. In the continuous cooling process, the carbon C/Mn element in the bainite diffuses to the untransformed austenite, resulting in carbon-rich austenite. In the subsequent cooling process, the carbon-rich undercooled austenite transforms into martensite, and a small amount of austenite is retained due to incomplete transformation. In other words, martensite–austenite (MA) constituents are formed. MA constituents are mainly distributed in strips between lath bainite and in blocks in granular bainite or austenite grain boundaries. Due to the high temperature heating of austenitizing and subsequent rapid cooling process, the non-equilibrium microstructure formed after quenching has a tendency to transform to the steady-state microstructure. The tempering heat treatment process provides thermodynamic conditions for the transformation of this microstructure, so as to obtain the hot bend pipe with excellent mechanical properties and dimensional stability. At the same time, the good microalloying design concept was adopted in the test materials. When the heating temperature was 950 °C, the undissolved carbonitrides, such as Nb/V/Ti, could prevent the growth of austenite grains through the mechanism of particle pinning grain boundaries, which established a good organizational foundation for the improvement in strength and toughness of X80 seamless bend pipe.

**Figure 4:**
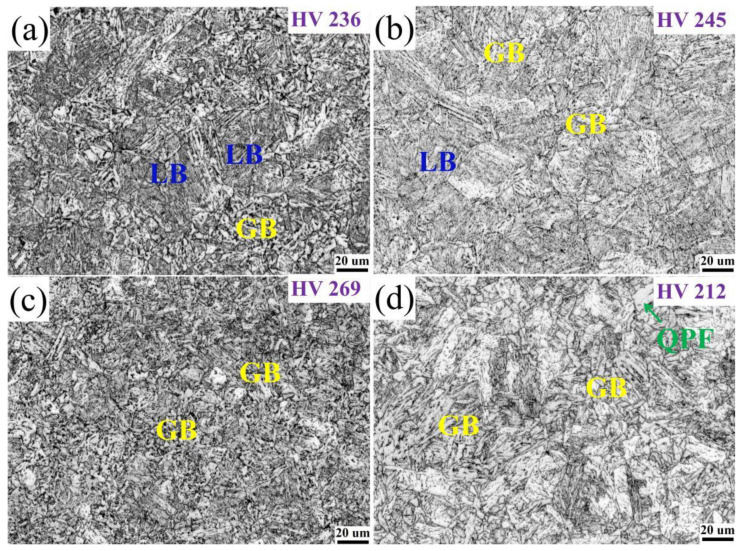
Optical micropraphs of heavy-wall seamless bend pipe steel after tempering at (**a**) 550 °C, (**b**) 600 °C, (**c**) 650 °C, and (**d**) 700 °C.

**Figure 5:**
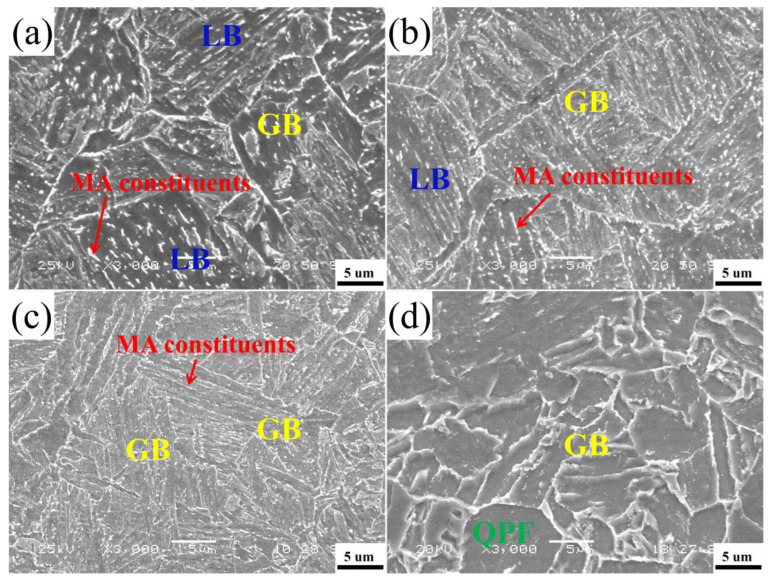
SEM micropraphs of heavy-wall seamless bend pipe steel after tempering at (**a**) 550 °C, (**b**) 600 °C, (**c**) 650 °C, and (**d**) 700 °C.

Figure 4 and Figure 5 show the metallographic structure and scanning electron microscope microstructure at different tempering temperatures after quenching. When the tempering temperatures were 550 °C and 600 °C, the microstructure of the investigated tempered steel was composed of lath bainite and granular bainite. As the tempering temperature increases, part of the lath bainite merges and widens, the number of granular bainite gradually increases, while the number of lath bainite decreases. The striped MA constituents decompose into dot or short rod shape. The final scenery is that part of the original austenite grain boundaries are clearly visible. The short rod-like MA constituents are distributed between the laths, and the dot-like MA constituents are distributed in the matrix of granular bainite. When the tempering temperature increases to 650 °C, the microstructure is almost entirely composed of granular bainite. The lath of ferrite is further widened and polygonalized, the lath bainite is transformed into granular bainite, and the effective grain size is refined. The MA constituents distributed at the grain boundary are completely decomposed, and the original austenite grain boundary is invisible. The size of the MA constituents in granular bainite becomes smaller, mainly distributed in the ferrite matrix or at the grain boundary in a dot shape, and some even decompose to disappear. When the tempering temperature increases to 700 °C, the microstructure is mainly composed of granular bainite and new quasi-polygonal ferrite (QPF). The MA constituents became smaller, mainly dispersed in the matrix tissue in a dot shape. Due to the high tempering temperature, the intensified atomic diffusion motion leads to the recrystallization of some microstructures [21].

#### 3.1.2. TEM Investigation

**Figure 6:**
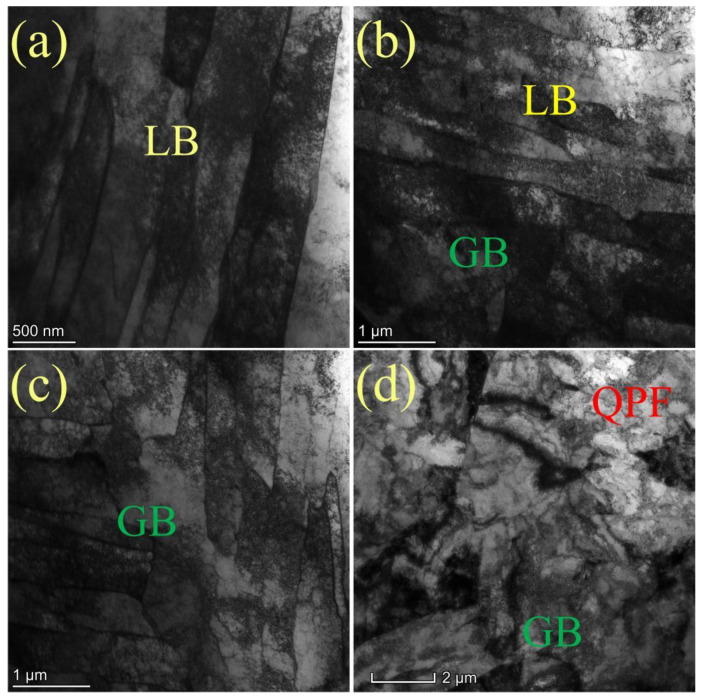
TEM micropraphs of heavy-wall seamless bend pipe steel after tempering at (**a**) 550 °C, (**b**) 600 °C, (**c**) 650 °C, and (**d**) 700 °C.

To further reveal the substructure of tempered steels at different temperatures, transmission electron microscopy was used for detailed observation. TEM investigation shows that, as shown in Figure 6, when the tempering temperature is 550 °C, the ferrite lath is slender, straight, and clear, showing parallel distribution, and there are high-density dislocations in the lath. When the tempering temperature is 600 °C, the elongated lath size becomes wider and some laths merge. When the tempering temperature reaches 650 °C, the lath structure is still maintained in the matrix, but the lath is further widened, and some laths are fused, recovered, and recrystallized, which makes the laths polygon. With the increase in tempering temperature, the width of elongated ferrite lath increases from 0.3–0.5 μm at 550 °C tempering to 0.42–1.28 nm at 650 °C tempering. When the tempering temperature was 700 °C, the microstructure of the bend pipe changed significantly, and polygonal ferrite with low dislocation density appeared. The number of lath bainite and granular bainite with high dislocation density decreased significantly, which was due to the recrystallization phenomenon of some microstructures caused by the intensified atomic diffusion movement at this temperature [22,23]. The laths are almost polygonal, and the matrix structure is blocky.

**Figure 7:**
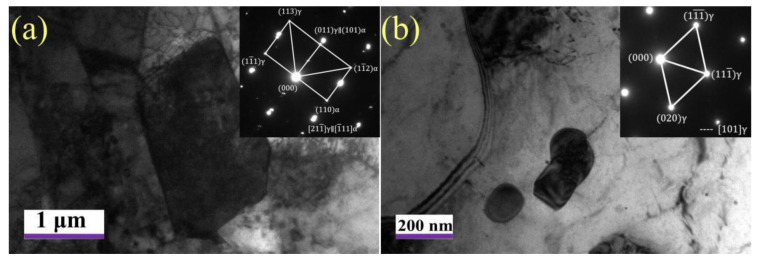
TEM micrographs of MA constituents tempered at 550 °C (**a**) and 650 °C (**b**).

MA constituents is the last transformation product in the cooling process, and its stability is the worst. Figure 7 further shows the morphology and size of the MA constituents. The results show that the large and blocky MA constituents are embedded in the ferrite matrix when tempered at 550 °C. With the increase in tempering temperature, the MA constituents gradually decompose when tempered at 650 °C, showing a granular distribution, and the size becomes significantly smaller.

### 3.2. Dislocation and Precipitation Investigation

#### 3.2.1. Dislocation Morphology and Precipitation Behavior by TEM

**Figure 8:**
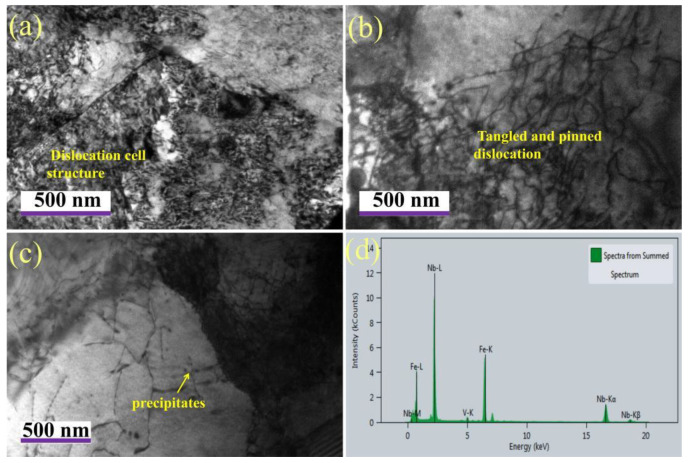
Dislocation morphology (**a**,**b**) and precipitation behavior (**c**,**d**) by TEM after tempering at 650 °C.

In the tempering process, the quenched structure has a tendency to change to the equilibrium structure, which also includes the disappearance, recombination and reorganization of dislocations. The speed of this process was mainly determined by the mobility of dislocations. Figure 8 shows the dislocation morphology of the sample after tempering at 650 °C. Most of the dislocations are mainly in the form of dislocated cell structures distributed in the ferrite matrix or entangled near the grain boundary, and some dislocations are relatively uniform distributed, parallel to each other and pinned by the precipitated phase. Wu et al. [23] shows that there are two kinds of dislocations in the mixed structure of lath bainite and granular bainite. One is the deformation dislocation formed in the deformation process of austenite region. Due to strain induction, Nb/Ti and other strong carbide-forming elements will generate a large number of carbides and precipitate and pin on such dislocations. These dislocations are basically inherited and still pinned during tempering. Another kind of dislocation in the lath is the phase change dislocation due to the volume effect during bainite transformation, which is relatively flat and not pinned by the precipitated phase. It is easy to disappear or slip to the grain boundary during tempering, but the matrix structure has no obvious change. Zhang et al. [20] suggested that the precipitates in the test steel include the strain-induced precipitates in the austenite stage, the phase boundary precipitates in the cooling stage, and the tempered precipitates in the tempering stage. However, in comparison, the particle size of the precipitates in the tempering stage is relatively small, which has a strong pinning effect and significantly enhances the precipitation strengthening effect. These tangled and pinned dislocations are relatively stable, which is conducive to maintaining the high strength of the material after tempering. Energy-dispersive X-ray spectroscopy analysis shows that these precipitates are mainly (Nb, V)(C, N). It can be seen that, although the second type of phase transformation dislocation is easy to slip during tempering, the high strength of the test steel is still maintained at 650 °C, mainly due to the precipitation strengthening effect during tempering, which is significantly greater than the softening caused by dislocation disappearance and microstructure evolution. On the other hand, the precipitated phase further pinned the dislocation, which enhanced the interaction between the precipitated phase and the dislocation [24,25,26,27]. With the further increase in tempering temperature to 700 °C, the precipitated phase grows rapidly and coarsens, and some dislocations begin to depin, which weakens the dislocation strengthening effect. However, TEM results show that the ferrite structure of the plate after high-temperature tempering does not completely disappear, which is consistent with the decrease in the strength of the sample tempered at 700 °C. This may be attributed to the fact that, even if there is a precipitation process during tempering, the softening caused by microstructure evolution due to dislocation disappearance cannot be compensated.

#### 3.2.2. Dislocation Density Analysis by XRD

**Figure 9:**
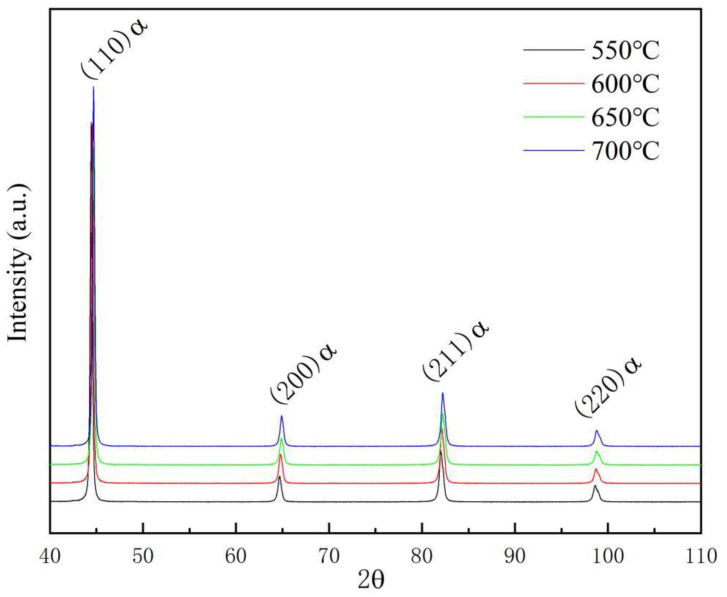
XRD patterns of heavy-wall seamless bend pipe steel after tempering.

Different samples were also investigated by XRD, and the typical XRD patterns are displayed in Figure 9. It is known that XRD peak profiles are the result of the instrument and tested samples. Hence, before calculating the dislocation density, the instrumental contribution to the peak broadening has subtracted from the row date [28,29,30]. Then, the dislocation density, *ρ*, was quantitatively calculated by the modified Williamson–Hall method, which can be expressed as the following Equation (1):

(1) $$\rho =\frac{14.4\varepsilon^{2}}{b^{2}}$$

where *ε* represents the non-uniform strain, and b represents the Burst’s vector of the dislocation in α-Fe (b = 0.248 nm) can be determined as the slope which was obtained by performing a linear regression of Δθ·cos θ/λ and 2 sin θ/λ of the first three peaks in XRD patterns. The dislocation densities of the samples tempered from 550 °C to 700 °C are 6.02 × 1014 mm/mm^3^, 4.74 × 1014 mm/mm^3^, 3.28 × 1014 mm/mm^3^, and 2.36 × 1014 mm/mm^3^, respectively. It can be concluded that dislocation density typically decreases when the tempering temperature increases.

### 3.3. Mechanical Properties

The mechanical properties of the investigated steel after different tempering temperatures are listed in Table 3, including yield strength, tensile strength, yield ratio, total elongation, and uniform elongation. It can be seen from the table that when the tempering temperature was 550, 600, 650, and 700 °C, the yield strength was 703, 709, 719, and 672 MPa, respectively. When tensile strength was 771, 778, 789, and 740 MPa, the total elongation was 20.5%, 22.5%, 22.5%, and 19%, respectively. The uniform elongation was 6.81%, 8.02%, 8.49%, and 5.59%, respectively. When tempered at 550–650 °C, the tensile strength and yield strength increased slightly with the increase ub tempering temperature, but no obvious increment was observed. When the tempering temperature continued to increase to 700 °C, the yield strength and tensile strength decreased significantly. When the tempering temperature was 650 °C, the material exhibited the best strength–plasticity, including the highest yield strength and the maximum total elongation, which is closely related to the generation and multiplication of a large number of dislocations, the interaction and entanglement effect between dislocations, and the interaction between dislocations and precipitates during deformation. After the uniform deformation stage, all samples can be observed with obvious necking behavior, and the final section shrinkage is 60–65% for all samples, which indicates that all samples are in a ductile failure mode.

## 4. Discussion

### 4.1. Effect of Tempering Treatment on Yield Behavior

**Figure 10:**
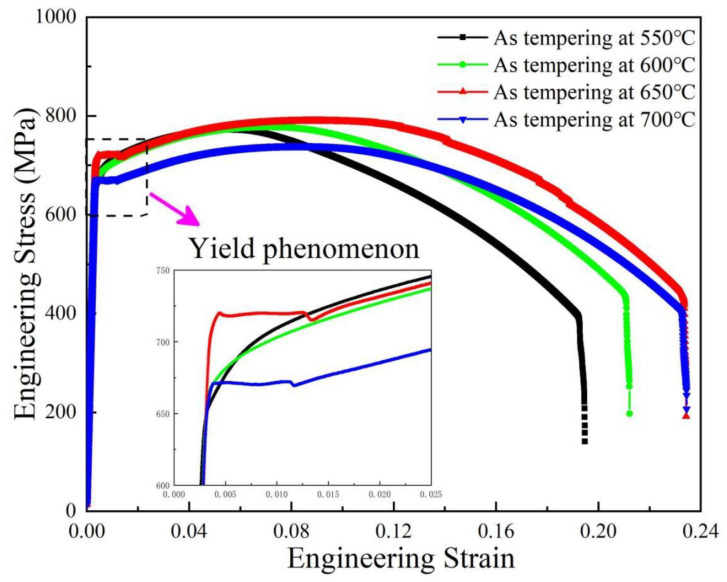
Tensile stress–strain curves after tempering at different temperatures.

Figure 10 shows the tensile stress–strain curves after tempering at different temperatures, and the locally enlarged pictures are inserted to clearly show the yield behavior of all samples. When the tempering temperature is 550 °C and 600 °C, the stress–strain curve shows a continuous yield behavior, namely, the round roof. When tempered at 650 °C and 700 °C, the stress–strain curves show the yield platform. The obvious transition point exists between elasticity and plasticity, indicating that its deformation behavior is different from the conventional continuous yield behavior. If the body-centered cubic metal contains a small amount of self-interstitial atoms, such as carbon and nitrogen, its stress–strain curve may indicate upper yield strength and a lower yield strength. Before the upper yield strength, there is elastic deformation, and after the upper yield, there is plastic deformation. Once the plastic deformation begins, the stress will have a drop, that is, to continue deformation at a low stress level (lower yield strength). At this time, the stress–strain curve is a horizontal line, and the work-hardening rate is zero. Then, the sample underwent work hardening, and the normal stress–strain curve was obtained. The reason for the up–down yield phenomenon is that the elastic interaction between the solute atoms in the C, N, and the dislocation generates the Cottrell atmosphere [31,32,33]. The dislocation movement pinned by the gas mass requires greater stress (or the dislocation density that can move is small, so that they need greater stress to move), which is the reason for the up–down yield strength. In the quenching process, when the shear transformation from austenite to bainite occurs, the residual tensile stress and the geometrically necessary dislocation density caused by volume expansion are generated. Residual stresses can be responsible for lower elastic limit, and geometric density can promote continuous yield behavior. However, in the tempering process, both high residual stress and high-density geometrically necessary dislocation can be reduced or annihilated. The continuous yield behavior is related to the high dislocation density at a low tempering temperature. When tempered at 550 °C and 600 °C, the geometry must link to high dislocation density. In the process of tensile deformation, the removable dislocation density is high, and the dislocation is easy to slip. Once the dislocation starts under the applied stress, it can continue to move under a relatively stable external force, showing a smooth elastic–plastic transition. When tempered at higher temperatures of 650 °C and 700 °C, the geometrically necessary dislocation density decreases, some movable dislocations slip, annihilate, and disappear, and the movable dislocation density decreases. In the process of tensile deformation, there are obvious upper and lower yield points, as well as obvious yield platforms on the stress–strain curve. This obvious yield phenomenon is related to the Cottrell atmosphere formed by C and N interstitial atoms at the dislocation. The Cottrell atmosphere has a strong pinning effect on the dislocation, and the dislocation must move out of the shackles of the atmosphere under a large external stress. This stress value is the upper yield point on the stress–strain curve. Once the dislocation gets rid of the pinning of the Cottrell atmosphere, it can move under a lower stress, which is the lower yield point on the stress–strain curve. At this time, as the strain increases, the stress value only produces small fluctuations, which is the yield platform on the curve.

### 4.2. Effect of Tempering Treatment on Uniform Plastic Deformation Behavior

One of the main tasks of studying plastic deformation behavior is to analyze the mechanical behavior and its characteristic properties in uniform plastic deformation stage. The Hollomon formula [34,35,36] describes the relationship between true stress and true strain in the deformation process of materials by the following Equation (2). The work-hardening exponent, n, is introduced into the field of plastic mechanics, which reflects the behavior characteristics of materials in the process of uniform plastic deformation:

(2) $$\sigma_{t}=K\cdot \varepsilon_{t}^{n}$$

where *σ_t_* and *ε_t_* are true stress and true strain, respectively; *n* is the work-hardening index; and *K* is the work-hardening coefficient. The work-hardening index can be obtained by taking logarithms on both sides of the Equation (3), as shown in the following:

(3) $$\ln\sigma_{t}=\ln K+n\cdot \ln\varepsilon_{t}\;n=\frac{d(\ln\sigma_{t})}{d(\ln\varepsilon_{t})}=\frac{\varepsilon_{t}}{\sigma_{t}}\cdot \frac{d\sigma_{t}}{d\varepsilon_{t}}$$

The work-hardening index, *n*, describes the sensitivity of the instantaneous true stress of material deformation to true strain from a mechanical point of view, and the value reflects the ability of the material to resist further plastic deformation. The derivation described in Equation (4) from the above equation shows that the work-hardening rate *dσ*/*dε* indicates the steepness of the true stress–true strain curve in the plastic deformation stage, but the relationship between *dσ*–*dε* and the work–hardening exponent, *n*, is not clear.

(4) $$\frac{d\sigma_{t}}{d\varepsilon_{t}}=n\times \frac{\sigma_{t}}{\varepsilon_{t}}$$

The local plastic deformation of structural materials during application mostly means failure or risk of failure. Therefore, it is of great practical significance to determine the uniform plastic strain of materials. When the uniform plastic strain is the maximum, the material necking occurs; however, the corresponding true stress–true strain curve does not reach the maximum, but into the non-uniform plastic deformation stage. The uniform plastic strain can be calculated by using the definition of true stress and true strain, as well as the Hollomon formula, which is expressed as:

(5) $$\sigma_{t}=\frac{d\sigma_{t}}{d\varepsilon_{t}}\;K\varepsilon_{t}=Kn\varepsilon_{t}{}^{n-1}\;\varepsilon_{t}=n$$

In summary, when the material necking occurs, the above formula holds, so the maximum uniform true strain is equal to the work-hardening index, *n*. Therefore, the work-hardening index, *n*, can measure the ability of metal materials to obtain uniform deformation by the work-hardening effect before necking.

**Figure 11:**
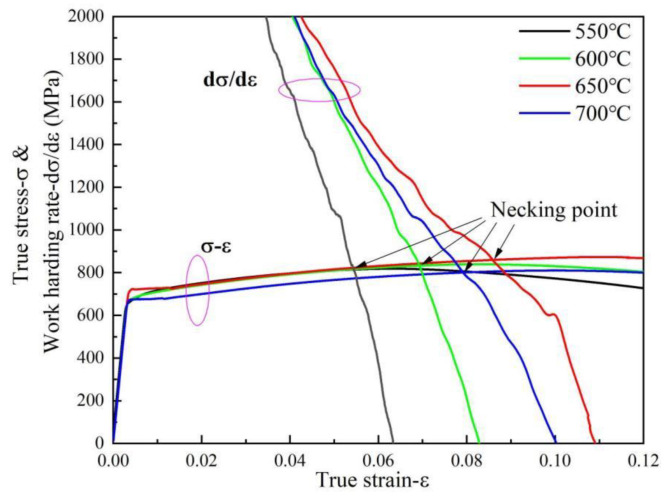
True stress and work-hardening rate curves versus true strain.

Figure 11 shows the true stress and work-hardening rate curves versus true strain diagrams of the steel investigated at different tempering temperatures. In the uniform deformation stage, in the range of 500–650 °C tempering, the work-hardening rate increases with the increase in tempering temperature, while the work-hardening rate of the 700 °C-tempered specimen is lower than that of 650 °C tempering. In the process of reducing the work-hardening rate from the highest point of plastic deformation to the necking point, the work-hardening rates of tempered specimens at 550 °C and 600 °C show a sharp and rapid decline. While 650 °C- and 700 °C-tempered specimens show a slow decline, especially when tempered at 650 °C, the work-hardening rate of the material decreases most slowly. The above results show that the specimen tempered at 650 °C exhibits the best work-hardening rate, indicating that it has a stronger ability to resist plastic deformation and delays the occurrence of necking, so it has the highest uniform elongation.

The deformation ability of pipeline steel is mainly concerned with the plasticity of pipeline material, that is, the material needs to have a higher hardening index *n* to obtain a larger uniform plastic elongation. In other words, the higher *n* value of the material is, the stronger the ability of the material to uniformly distributing strain is due to the work-hardening effect. Whether the processing performance or the use of performance requirements, it is hoped that the material has a strong work-hardening characteristics, so as to obtain a higher uniform deformation ability, which is the reason to study the plastic deformation characteristics of metal materials.

### 4.3. Effect of Tempering Treatment on Work-Hardening Behavior

**Figure 12:**
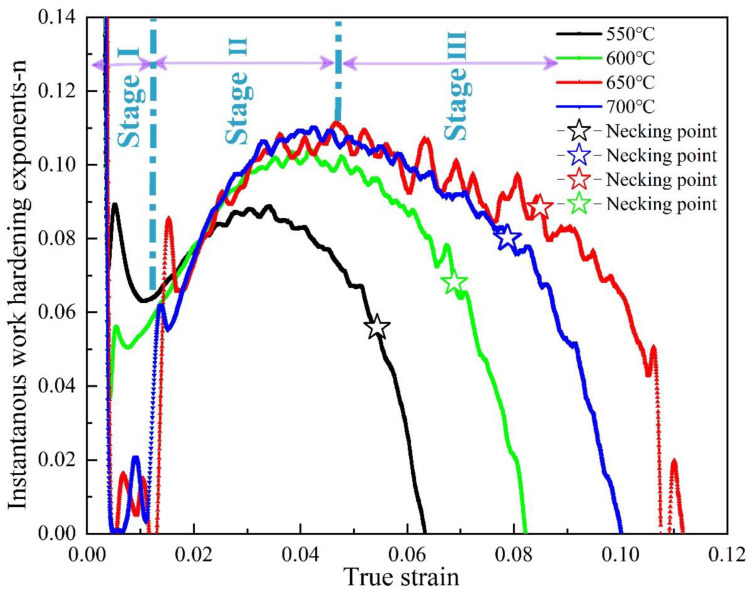
Instantaneous work-hardening exponent, *n*, as a function of true strain.

Different tempering temperatures not only affect the yield behavior and yield strength of the material, but also affect the work-hardening ability of the material [37]. The instantaneous work-hardening exponent, n, can more accurately describe the sensitivity of the work-hardening ability of materials to strain and the characteristics of the hardening stage during the tensile process [38,39,40,41,42]. The instantaneous work-hardening exponent of the investigated steel at different tempering temperatures is calculated according to Equation (4), as shown in Figure 12. The work-hardening behavior of materials can be divided into three stages: (1) the rapid hardening stage—the initial instantaneous value n is very high, but with the increase in true strain n, the value decreases rapidly; (2) the stable hardening stage—with the increase in true strain, the n value continues to increase; and (3) the hardening exponents decline stage—as the deformation stage, the instantaneous n value decreased rapidly. In the rapid hardening process of the first stage, when the tempering temperature is 650 °C and 700 °C, the instantaneous work-hardening index is close to zero, and there is no rapid hardening process of the first stage of the material. This is due to the yield phenomenon (the generation of yield platform) without work hardening in the initial stage of deformation after elastic deformation. The rapid hardening during tempering at lower temperatures is attributed to the movable dislocations to the density. Once these dislocations start under applied stress, they will interact with each other to produce pile-up, resulting in a certain work-hardening ability. In the second stage of stable hardening, the n values of the 650 °C- and 700 °C-tempered specimens were significantly higher than those of the 550 °C- and 600 °C-tempered specimens at low temperature, while the specimen tempered at 650 °C showed the maximum work-hardening index. With the increase in tempering temperature, the instantaneous work-hardening index, n, increases. After yielding, a large number of dislocations proliferate and move along the slip surface in the sample tempered at high temperature with low original dislocation density. The interaction of dislocations, the interaction between dislocations and carbonitrides, and the accumulation of dislocations at grain boundaries make the movement of dislocations gradually difficult, which is the reason for the high work-hardening effect at this stage. At this stage, the number of dislocations in the low-temperature tempered sample with high dislocation density is less than that in the high-temperature tempered sample, and there are enough dislocations to generate slip deformation. The higher instantaneous work-hardening n value of the sample tempered at high temperature is related to the precipitation-strengthening effect during tempering. The combined effect of these two aspects means that the samples tempered at a high temperature show a higher work-hardening index, *n*, especially the sample tempered at 650 °C which shows the maximum instantaneous work-hardening index. The instantaneous work-hardening index of the 550 °C-tempered specimen is lower than that of the 600 °C-tempered specimen, which may be due to the residual stress generated during the transformation of austenite to bainite, which has not been completely eliminated during tempering, resulting in large internal stress in the material. In addition, the larger internal stress promotes the easier slip of dislocations when tempered at lower temperatures, so the work-hardening index of the sample tempered at 550 °C is lower than that tempered at 600 °C. As the true strain continues to increase during tensile deformation, the instantaneous work-hardening index reaches the maximum value, and then begins to decrease, which is attributed to the annihilation of a large number of dislocations. More and more dislocations accumulate near the grain boundary, forming a strong stress concentration at the grain boundary. More and more dislocations pile up near the grain boundary, forming a strong stress concentration at the grain boundary. The obstacles in front of the plugged dislocation group were destroyed under the high stress concentration, so that the high stress field of the plugged dislocation group is relaxed. Furthermore, when the stress concentration caused by dislocation pile-up exceeds the grain boundary strength, holes and microcracks are generated on the grain boundary. Dislocations disappear in large quantities at the grain boundary defects, the dislocation density decreases rapidly, and the work-hardening ability decreases significantly. At the same time, it can be observed that, in the third stage, the instantaneous work-hardening index of the material forms a transition point at the necking point. In the uniform deformation stage before the necking point, the instantaneous work-hardening index decreases slowly, and after the necking point, the material enters the non-uniform deformation stage. At this time, the instantaneous work-hardening index decreases sharply, indicating that the engineering stress of the material reaches the strength limit when necking occurs, but the true stress continues to increase, and the instantaneous work-hardening index decreases rapidly. This is mainly related to the serious plastic damage of the material caused by the formation, expansion, and connection of a large number of micro-pores when necking occurs. When the instantaneous work-hardening index drops to zero, it indicates that the true stress–true strain curve of the material begins to decrease with the increase in true strain. This is mainly due to the fact that, after the necking occurs, the local large deformation causes the material to shift to other parts in the form of dislocation, which reduces the tensile force required for the continuous deformation of the specimen, and finally leads to the plastic fracture of the specimen at the necking [40,41,42].

## 5. Conclusions

When the investigated steel was tempered from 550 °C to 650 °C after quenching, with the increase in tempering temperature, the microstructure gradually changed from lath bainite to granular bainite. When the tempering temperature reaches 700 °C, a large number of polygonal ferrite appears.

With the increase in tempering temperature, the dislocation density of the sample decreases, but the precipitated phase particles gradually increase. Samples tempered at 650 °C showed the highest yield strength, which was related to the tangled dislocation cell structure, pinned dislocation, and the precipitation of carbon nitride.

The formation of yield platforms in the samples tempered at 650 °C and 700 °C was related to the decrease in mobile dislocation density and the strong pinning effect of Cottrell atmosphere on dislocation.

The excellent uniform elongation of the sample tempered at 650 °C is due to the high work-hardening rate and instantaneous work-hardening index, which is the result of dislocation multiplication, dislocation–dislocation interaction, and dislocation–precipitates interaction.

## Figures and Tables

**Table 1:** Chemical elements of the X80 heavy-wall seamless pipeline steel (wt%).

C	Mn	Si	P	S	Ni	Cr	V	Ti	Mo	Nb
0.10	1.06	0.22	0.005	0.002	1.0	0.29	0.07	0.003	0.39	0.03

**Table 2:** Heat induction bending parameters of heavy-wall X80 × D1422 mm heat induction seamless bend.

Heating Temperature (°C)	Push Speed (mm/s)	Cooling Water Flow (m^3^/h)	Bending Angle (°)
1050	0.3	50	4

**Table 3:** Mechanical properties of studied steel after different tempering treatments.

Tempering Temperature (°C)	Yield Strength (MPa)	Ultimate Tensile Strength (MPa)	Yield Strength Ratio	Total Elongation (%)	Uniform Elongation (%)
550	703 ± 6	771 ± 4	0.91	20.5 ± 0.2	6.81 ± 0.20
600	709 ± 5	778 ± 5	0.91	22.5 ± 0.3	8.02 ± 0.16
650	719 ± 5	789 ± 4	0.91	22.5 ± 0.2	8.49 ± 0.15
700	672 ± 4	740 ± 4	0.90	19.0 ± 0.3	5.59 ± 0.21

## Data Availability

Data sharing is not applicable to this article.

## References

[1] Sharma S.K., Maheshwari S. (2017). A review on welding of high strength oil and gas pipeline steels. J. Nat. Gas Sci. Eng..

[2] Zhang X., Gao H., Zhang X., Yang Y. (2012). Effect of volume fraction of bainite on microstructure and mechanical properties of X80 pipeline steel with excellent deformability. Mater. Sci. Eng. A.

[3] Ning J., Yu Z.-S., Sun K., Hu M.-J., Zhang L.-X., Zhang Y.-B., Zhang L.-J. (2021). Comparison of microstructures and properties of X80 pipeline steel additively manufactured based on laser welding with filler wire and cold metal transfer. J. Mater. Res. Technol..

[4] Tu X., Shi X., Yan W., Li C., Shi Q., Shan Y., Yang K. (2022). Tensile deformation behavior of ferrite-bainite dual-phase pipeline steel. Mater. Sci. Eng. A.

[5] Jorge J.C.F., Souza L.F.G.D., Mendes M.C., Bott I.S., Araújo L.S., Santos V.R.D., Rebello J.M.A., Evans G.M. (2021). Microstructure characterization and its relationship with impact toughness of C–Mn and high strength low alloy steel weld metals-a review. J. Mater. Res. Technol..

[6] Bott I.d.S., De Souza L.F.G., Teixeira J.C.G., Rios P.R. (2005). High-strength steel development for pipelines: A brazilian perspective. Metall. Mater. Trans. A.

[7] Pamnani R., Karthik V., Jayakumar T., Vasudevan M., Sakthivel T. (2016). Evaluation of mechanical properties across micro alloyed HSLA steel weld joints using Automated Ball Indentation. Mater. Sci. Eng. A.

[8] Vervynckt S., Verbeken K., Lopez B., Jonas J.J. (2012). Modern HSLA steels and role of non-recrystallisation temperature. Int. Mater. Rev..

[9] Jia Z., Misra R.D.K., O’Malley R., Jansto S.J. (2011). Fine-scale precipitation and mechanical properties of thin slab processed titanium–niobium bearing high strength steels. Mater. Sci. Eng. A.

[10] Hu J., Du L.-X., Xie H., Gao X.-H., Misra R.D.K. (2014). Microstructure and mechanical properties of TMCP heavy plate microalloyed steel. Mater. Sci. Eng. A.

[11] Hu J., Du L.-X., Wang J.-J., Xie H., Gao C.-R., Misra R.D.K. (2013). Structure–mechanical property relationship in low carbon microalloyed steel plate processed using controlled rolling and two-stage continuous cooling. Mater. Sci. Eng. A.

[12] Anijdan S.H.M., Sediako D., Yue S. (2012). Optimization of flow stress in cool deformed Nb-microalloyed steel by combining strain induced transformation of retained austenite, cooling rate and heat treatment. Acta Mater..

[13] Lee S.H., Kim K.H., Van D., Nam S. (2020). Effect of C-Mn ratio on the maximum hardness and toughness in TMCP steels with an identical carbon equivalent. J. Mater. Res. Technol..

[14] Wang B., Lian J. (2014). Effect of microstructure on low-temperature toughness of a low carbon Nb–V–Ti microalloyed pipeline steel. Mater. Sci. Eng. A.

[15] Wang X., Liao B., Wu D.-Y., Han X.-L., Zhang Y.-S., Xiao F.-R. (2014). Effects of Hot Bending Parameters on Microstructure and Mechanical Properties of Weld Metal for X80 Hot Bends. J. Iron Steel Res. Int..

[16] Wang X., Xiao F.-R., Fu Y.-H., Chen X.-W., Liao B. (2011). Material development for grade X80 heavy-wall hot induction bends. Mater. Sci. Eng. A.

[17] Zhou T., Yu H., Hu J., Wang S. (2014). Study of microstructural evolution and strength–toughness mechanism of heavy-wall induction bend pipe. Mater. Sci. Eng. A.

[18] Wang X., Zhou J., Liang Q. (2014). Multi-objective Optimization of Medium Frequency Induction Heating Process for Large Diameter Pipe Bending. Procedia Eng..

[19] Wang L., Wang B., Zhou P. (2018). Misorientation, grain boundary, texture and recrystallization study in X90 hot bend related to mechanical properties. Mater. Sci. Eng. A.

[20] Zhang X.Y., Tian C.C., Gao H.L., Liu Y.L., Zhang X.Q. (2012). Effects of tempering temperature on microstructures and properties of X80 grade heat-bending bend. Trans. Mater. Heat Treat..

[21] Xie Z.J., Yuan S.F., Zhou W.H., Yang J.R., Guo H., Shang C.J. (2014). Stabilization of retained austenite by the two-step intercritical heat treatment and its effect on the toughness of a low alloyed steel. Mater. Des..

[22] Zhou W.H., Wang X.L., Venkatsurya P.K.C., Guo H., Shang C.J., Misra R.D.K. (2014). Structure–mechanical property relationship in a high strength low carbon alloy steel processed by two-step intercritical annealing and intercritical tempering. Mater. Sci. Eng. A.

[23] Wu H.B., Shang C.J., Yuan S.Q., Yang S.W., Wang X.M., He X.L. (2004). The Tempering Microstructures and Mechanical Properties in an Ultra-fine Low Carbon Bainitic Steel. Trans. Mater. Heat Treat..

[24] Guo A., Misra R.D.K., Xu J., Guo B., Jansto S.G. (2010). Ultrahigh strength and low yield ratio of niobium-microalloyed 900 MPa pipeline steel with nano/ultrafine bainitic lath. Mater. Sci. Eng. A.

[25] Zaefferer S., Ohlert J., Bleck W. (2004). A study of microstructure, transformation mechanisms and correlation between microstructure and mechanical properties of a low alloyed TRIP steel. Acta Mater..

[26] Sauvage X., Wilde G., Divinski S.V., Horita Z., Valiev R.Z. (2012). Grain boundaries in ultrafine grained materials processed by severe plastic deformation and related phenomena. Mater. Sci. Eng. A.

[27] Craven A.J., He K., Garvie L.A.J., Baker T.N. (2000). Complex heterogeneous precipitation in titanium–niobium microalloyed Al-killed HSLA steels—I. (Ti,Nb)(C,N) particles. Acta Mater..

[28] Lin S., Wang D., Li C., Liu X., Di X., Jiang Y. (2018). Effect of cyclic plastic deformation on microstructure and mechanical properties of weld metals used for reel-lay pipeline steels. Mater. Sci. Eng. A.

[29] Cong Z., Murata Y. (2011). Dislocation Density of Lath Martensite in 10Cr-5W Heat-Resistant Steels. Mater. Trans..

[30] Williamson G.K., Smallman R.E. (1956). III. Dislocation densities in some annealed and cold-worked metals from measurements on the X-ray debye-scherrer spectrum. Philos. Mag..

[31] Waseda O., Veiga R.G.A., Morthomas J., Chantrenne P., Becquart C.S., Ribeiro F., Jelea A., Goldenstein H., Perez M. (2017). Formation of carbon Cottrell atmospheres and their effect on the stress field around an edge dislocation. Scripta Mater..

[32] Kirchheim R. (2007). Reducing grain boundary, dislocation line and vacancy formation energies by solute segregation. I. Theoretical background. Acta Mater..

[33] Zecevic M., Knezevic M. (2015). A dislocation density based elasto-plastic self-consistent model for the prediction of cyclic deformation: Application to AA6022-T4. Int. J. Plast..

[34] Beyerlein I.J. (2008). Plastic behavior of metals in reverse straining after large pre-strains. Mater. Sci. Forum.

[35] Samuel F.H. (1987). Tensile stress-strain analysis of dual-phase structures in an Mn-Cr-Si steel. Mater. Sci. Eng..

[36] Li C.-N., Ji F.-Q., Yuan G., Kang J., Misra R.D.K., Wang G.-D. (2016). The impact of thermo-mechanical controlled processing on structure-property relationship and strain hardening behavior in dual-phase steels. Mater. Sci. Eng. A.

[37] Essmann U., Mughrabi H. (2006). Annihilation of dislocations during tensile and cyclic deformation and limits of dislocation densities. Philos. Mag..

[38] Mughrabi H. (1983). Dislocation wall and cell structures and long-range internal stresses in deformed metal crystals. Acta Metall..

[39] Kamikawa N., Sato K., Miyamoto G., Murayama M., Sekido N., Tsuzaki K., Furuhara T. (2015). Stress–strain behavior of ferrite and bainite with nano-precipitation in low carbon steels. Acta Mater..

[40] Wang Y., Sun J., Jiang T., Sun Y., Guo S., Liu Y. (2018). A low-alloy high-carbon martensite steel with 2.6 GPa tensile strength and good ductility. Acta Mater..

[41] Ye W., Li Y., Wang F. (2009). The improvement of the corrosion resistance of 309 stainless steel in the transpassive region by nano-crystallization. Electrochim. Acta.

[42] Luo Z.F., Liang Y.L., Long S.L., Jiang Y., Wu Z.L. (2017). Effects of ultra-refine grain and micro-nano twins on mechanical properties of 51CrV4 spring steel. Mater. Sci. Eng. A.

